# Nutlin-3a Decreases Male Fertility via UQCRC2

**DOI:** 10.1371/journal.pone.0076959

**Published:** 2013-10-09

**Authors:** Kamla Kant Shukla, Woo-Sung Kwon, Md Saidur Rahman, Yoo-Jin Park, Young-Ah You, Myung-Geol Pang

**Affiliations:** Department of Animal Science & Technology, Chung-Ang University, Anseong, Gyeonggi-do, South Korea; University Hospital of Münster, Germany

## Abstract

Ubiquinol-cytochrome-c reductase core protein 2 (UQCRC2) is a component of ubiquinol-cytochrome c reductase complex that is known to correlate with male fertility via spermatogenesis. Simultaneously, nutlin-3a is a small molecule antagonist of mouse double minute 2 repressor (MDM2), activate p53 and induce apoptosis responsible for spermatogenesis. To date, however there are no known effects of nutlin-3a on reproduction. Therefore, present study was designed to investigate the effect of nutlin-3a on male fertility via UQCRC2. In this *in vitro* trial with mice spermatozoa, we utilized CASA, CTC staining, ATP assay, western blotting, and IVF to measure the main study outcome. The short-term exposure of spermatozoa in nutlin-3a decreases sperm motion kinematics, intracellular ATP production, capacitation, the acrosome reaction, UQCRC2, and tyrosine phosphorylation (TYP) of sperm proteins in a dose-dependent manner. Notably, the decreased UQCRC2 and TYP were associated with reduced sperm kinematics, ATP production, and capacitation, which ultimately led to adverse effects on male fertility such as poor fertilization rates and embryo development. Thus, nutlin-3a may be considered as a potential male contraceptive agent due to its ability to decrease fertility secondary to changes in overall sperm physiology and embryonic development. However, the results of this preliminary study have to be confirmed by additional independent trial.

## Introduction

Physiologically normal spermatozoa are essential for successful fertilization of the female gamete both *in vitro* and *in vivo*, as well as for normal embryonic development. These processes require that the spermatozoa possess a specific set of proteins. Since mature spermatozoa are silent during both transcriptional and translational events and are therefore incapable of protein synthesis, the presence or absence of specific antagonist molecules is an ideal mechanism by which overall sperm function may be maximized or minimized.

 Protein ubiquitination alters protein activity and induces proteolysis via the 26S proteasome [1]. p53, a protein associated with ubiquitination, is an essential protein present during spermatogenesis that causes DNA damage and eliminates spermatogenic cells. TheMDM2 repressor can potentially bind the N-terminus of p53, and in turn promote ubiquitination which allows the protein to move from the nucleus to the cytoplasm of the cell, where p53 can then be degraded by cytoplasmic proteasomes [2]. A lack of adequate concentrations of p53 may result in atypical spermatogenesis and increased morphological abnormalities or spermatozoa containing damaged DNA [3]. Recently, it was reported that expression of ubiquinol-cytochrome c reductase core protein II (UQCRC2) in spermatozoa was correlated with male fertility [4].

 Tyrosine phosphorylation (TYP) of sperm proteins involved in the regulation of mammalian sperm cyclic adenosine monophosphate (cAMP) metabolism affect the ability to trigger sperm capacitation and the acrosome reaction. These fundamentally important events serve to increase the concentration of intracellular second messenger [5]. It has also been suggested that ATP increases in vitro fertilization in mouse spermatozoa without affecting the typical alterations of ATP-dependent protein tyrosine phosphorylation and acrosomal exocytosis in capacitated spermatozoa [6]. Simultaneously, [Ca^2+^]_i_ play an important role in the regulation of capacitation and the acrosome reaction, an exocytotic event that allows sperm to penetrate the zona pellucida and fuse with the oocyte plasma membrane towards fertilization [7].

 The pathogenesis of many human diseases has been largely linked to defects in the ubiquitin proteasome signaling (UPS) pathway [8]. However, ubiquitin dependent proteolysis of the p53 and UQCRC2 regulatory proteins is regulated by UPS via numerous cellular mechanisms. For example, nutlin-3a inhibits the binding of MDM2 to p53, thereby enhancing the activity of p53 and providing protection from ubiquitin-mediated proteasomal degradation [9]. Moreover, activation of the nutlin-3a-mediated p53 pathway leads to apoptosis in various malignancies including multiple myeloma [9]. Tumor suppressor activity of nutlin-3a both *in vitro* and *in vivo* has been shown by Ohnstad et al. [10], though its therapeutic value in the field of infertility has yet to be studied. In this study, we explored the cellular outcomes of using nutlin-3a as an antagonist to decrease the reproductive potential of mouse spermatozoa and to investigate the molecular mechanisms of sperm dysfunction associated with UQCRC2, TYP, ATP generation, decreased fertilization, and poor embryonic development.

## Materials and Methods

### Ethical statement

 All animals were maintained and used under protocols approved by the Institutional Animal Care and Ethical Committee of Chung-Ang University, Seoul, Republic Korea, according to the Guide for Care, Treatment, and Use of Laboratory Animals.

### Reagents

 All reagents were obtained from Sigma-Aldrich (St Louis, MO, USA). Modified Tyrode's medium (mT6) was freshly prepared before each experiment according to methods reported by Quinn et al. [11]. Briefly, the basic medium (BM) mT6 was composed of 97.84 mM NaCl, 1.42 mM KCl, 0.47 mM MgCl_2_·H_2_O, 0.36 mM NaH_2_PO_4_·H2O, 5.56 mM D-glucose, 25 mM NaHCO_3_, 1.78 mM CaCl_2_·H_2_O, 24.9 mM Na-lactate, 0.47 mM Na-pyruvate, and 50 µg/ml gentamycin. Bovine serum albumin (BSA; 4 mg/ml) was added to the BM for sperm capacitation. A stock solution of nutlin-3a was diluted with dimethyl sulfoxide and stored in a plastic container at -20°C. The nutlin-3a reactions were performed in a safety cabinet at culture room settings. Stock solution was added to the BM media to final molar concentrations of 1, 10, and 100 µM.

### Preparation and treatment of spermatozoa

 ICR mice (8–12 weeks of age) were used to prepare the mouse sperm suspension (Nara Biotech®, Seoul, Korea). Spermatozoa were collected based on previously described methods [12,13]. Briefly, the cauda epididymis of each mouse was separated and the surrounding fat was carefully removed. Each sample was placed on a piece of filter paper to remove excess fluid and fat. The cauda epididymis was excised using a surgical blade, and spermatozoa were released into BM containing 0.4% BSA in 35 mm sterile cell culture dishes. The spermatozoa were then incubated for 12 min in presence of 5% CO_2_ at 37°C. The sperm suspension was subsequently incubated in BM supplemented with 0, 1, 10, and 100 µM of nutlin-3a for an additional 90 min in air at 37°C for capacitation, respectively. 

### Computer-assisted sperm analysis (CASA)

 A CASA system (SAIS Plus version 10.1, Medical Supply, Seoul, Korea) was employed for analysis of sperm motion. Briefly, 10 µl of each sample was placed in a Makler chamber (Makler, Haifa, Israel). The filled chamber was then placed on a 37°C heated stage. Using a 10 x objective in phase contrast mode, the image was relayed, digitized, and analyzed by SAIS. The movement of at least 250 sperm cells was recorded from random fields (>5) in each sample. User-defined settings were as follows: frames acquired, 20; frame rate, 30 Hz; minimum contrast, 7; minimum size, 5; low/high size gates, 0.4-1.5; low/high intensity gates, 0.4-1.5; non-motile head size, 16; and non-motile brightness, 14.

### Combined Hoechst 33258/chlortetracycline fluorescence assessment of capacitation status (H33258/CTC)

 We used a dual staining method to determine capacitation status, as previously described [12–14]. Briefly, 135 µl of each treated spermatozoa sample was supplemented with 15 µl of H33258 solution (10 µl H33258/ml DPBS) and incubated for 2 min at room temperature (RT). Excess dye was removed by layering the mixture over 250 µl of 2% (w/v) polyvinylpyrrolidone in DPBS. After centrifuging at 100 x g for 2.5 min, the supernatant was discarded and the pellet was resuspended with 100 µl of DPBS and chlortetracycline fluorescence (CTC) solution (750 mM CTC in 5 µl buffer: 20 mM Tris, 130 mM NaCl, and 5 mM cysteine). Capacitation status was assessed using a Nikon microphot-FXA (Tokyo, Japan) under epifluorescence illumination using UV BP 340- 380/LP 425 and BP 450-490/LP 515 excitation/emission filters for H33258 and CTC, respectively. The spermatozoa were classified as live non-capacitated (F pattern, bright green fluorescence distributed uniformly over the entire sperm head, with or without a stronger fluorescent line at the equatorial segment), live capacitated (B pattern, green fluorescence over the acrosomal region and a dark post acrosome), or live acrosome reacted (AR pattern, sperm showing a mottled green fluorescence over the head, green fluorescence only in the post acrosomal region or no fluorescence over the head) [15]. Two slides per sample were evaluated, with at least 400 spermatozoa on each slide.

### Intracellular Calcium ([Ca^2+^]_i_) Ion Concentration

 Samples were incubated with 5 µM Fura-2 acetoxymethyl ester (AM) (Molecular Probes) that added 60 min after the onset of capacitation at 37°C in an atmosphere of 5% CO_2_ in air [12,13]. After incubation samples were centrifuged at 200 x g for 10 min and resuspended with DPBS. The suspension was illuminated with two excitation wavelengths (340 nm and 380 nm) and the emitted fluorescence was measured at 510 nm. [Ca^2+^]_i_ was calculated as the ratio of fluorescence from excitation at 340 nm to that at 380 nm. Fluorescence signals were detected with microplate fluorometer (Gemini Em; Molecular Devices Corporation, Sunnyvale, CA, USA) and calculated with SoftMax Pro 5 (Molecular Devices). Fluorescence ratio of all treatments was calculated by ratio of dDAVP treatment/ratio of control, respectively.

### Measurement of intracellular ATP

 Quantitative measurement of intracellular ATP production was performed using an ATP Bioluminescence Assay Kit CLS II (Roche Molecular Biochemicals, GmbH, Germany) according to the manufacturer’s instructions. Briefly, the cells were diluted to a concentration of 105-108 cells/µl (25 µl) in a 96-well plate. Equal volumes of cell lysis reagent were added to each well and incubated at RT for 5 min. Finally, ATP dilutions in a 50 µl volume were assayed with 50 µl luciferase reagent in a 96-well plate. Luminescence was measured by a luminometer (GEMINI EM, Molecular Devices Corporation) and calculated using SoftMax Pro 5 software (Molecular Devices Corporation).

### Western blotting of sperm proteins

 Each sample was washed twice with DPBS by centrifugation at 10,000x g for 3 min after incubation. Sperm pellets were resuspended in Laemmli sample buffer (63 mM 1 Tris, 10% glycerol, 10% sodium dodecyl sulfate, 5% bromophenol blue) containing 5% 2-mercaptoethanol and incubated for 10 min at RT. Finally, the supernatants were separated by centrifugation at 12,000x g for 10 min and boiled for 3 min at 100°C. Samples were subjected to SDS-polyacrylamide gel electrophoresis using a 12% mini-gel system (Amersham, Piscataway, NJ, USA), and the separated proteins were transferred to a membrane. UQCRC-2 was detected by incubation with monoclonal anti-UQCRC-2 goat antibody (Abcam, Cambridge, UK) diluted with blocking solution (1:2,000) for 2 h at RT. Next, α-tubulin was detected by incubation with monoclonal anti-a-tubulin mouse antibody (Abcam) diluted with 5% blocking agent (1:2,000) for 2 hat RT. The membranes were then incubated with horseradish peroxidase (HRP)-conjugated anti-rabbit IgG (Abcam) for 1 hat RT. Tyrosine phosphorylation proteins from the treated spermatozoa were immunodetected by first incubating the membranes with a HRP conjugated monoclonal anti-phosphotyrosine mouse antibody (pY20; Abcam) diluted 1:5,000 overnight at 4°C. Proteins on the membrane were visualized using an enhanced chemiluminescence (ECL) technique. All bands were scanned with a GS-800 Calibrated Imaging Densitometer (Bio-Rad, Fremont, CA, USA) and analyzed using Quantity One software (Bio-Rad, Hercules, CA, USA). The density of the bands was quantified according to the α-tubulin ratio (UQCRC2/α-tubulin or tyrosine phosphorylated protein/α-tubulin).

### In vitro fertilization

 Female B6D2F1/CrljOri mice (8-12 weeks of age) were purchased from Nara Biotech (Seoul, Korea). These mice were superovulated with a 5 IU i.p injection of pregnant mare serum gonadotropin and a 5 IU i.p. injection of human chorionic gonadotropin given 48 h later. Female mice were sacrificed 15 h after the second injection, and cumulus-oocyte complexes (COCs) were collect from the ampullae of the oviducts and placed in DPBS in a sterile culture dish. COCs were placed in 50 μl of BM supplemented with 10% FBS in mineral oil and then incubated for 1 h prior to insemination. The COCs were then incubated with washed 1 x 10^6^/ml motile spermatozoa with BM for 6h at 37°C under 5% CO_2_ in air. Eighteen hours after insemination, fertilization was assessed by determining the number of two-cell embryos in each sample. Each two-cell oocyte was placed into a new culture dish with 50 µl BM supplemented with 0.4% BSA for 5 days at 37°C under 5% CO_2_ in air. All embryos were utilized, including those that developed to the blastocyst stage and those that remained at the two-cell stage.

### Statistical analysis

 One-way ANOVA of SPSS (v. 12.0; Chicago, IL, USA) was utilized to analyzed the data with a Tukey's test to locate differences. This test compares responses within replicates; for a significant difference to be obtained, a consistent and reasonable magnitude is required between control and treated samples with nutiln-3a. *p*<0.05 was considered statistically significant and numerical data are expressed as mean ± SEM.

## Results

### Effects of nutlin-3a on sperm motion kinematics

 A computer-assisted sperm analysis (CASA) system was employed to monitor the sperm motion kinematics after 90 min incubation in basic media (mT6) in the presence of nutlin-3a (0, 1, 10, and 100 µM). Each spermatozoa sample treated with increasing concentrations of nutlin-3a displayed decreases in the motion parameters measured (percentage of motile spermatozoa (MOT), percentage of hyper-activated motile spermatozoa (HYP), curvilinear velocity (VCL), straight line velocity (VSL), average path velocity (VAP), linearity (LIN), straightness (STR), wobble (WOB), and lateral head displacement (ALH)) in a dose-dependent manner (Table 1). Consequently, spermatozoa treated with the highest dose of nutlin-3a (100μM) showed statistically significant decreases compared with the other samples (*p*<0.05)***.***

**Table 1:** Effect of nutlin-3a on sperm motion parameters.

	Motion kinematics								
Treatments	MOT (%)	HYP (%)	VCL (μm/s)	VSL (μm/s)	VAP (μm/s)	LIN (%)	STR (%)	WOB (%)	ALH (μm)
Control	71.60 ± 2.54^a^	14.55 ± 3.14^a^	144.93 ± 5.82^a^	73.08 ± 3.17^a^	75.12 ± 3.07^a^	50.25 ± 1.78^a^	96.41 ± 1.27^a^	51.86 ± 1.18^a^	6.08 ± 0.2^a^
1µM Nutlin-3a	69.01 ± 3^a,b^	12.69 ± 1.98^a,b^	139.14 ± 2.14^a^	68.59 ± 2.77^a^	72.29 ± 2.23^a^	49.33 ± 2.05^a^	94.63 ± 1.27^a^	51.97 ± 1.67^a^	6.08 ± 0.11^a^
10µM Nutlin-3a	62.03 ± 1.2^b^	5.84 ± 0.42^b,c^	119.32 ± 2.47^b^	56.7 ± 2.73^b^	63.39 ± 2.29^b^	38.8 ± 1.55^a^	91.87 ±1.6^a^	51.47 ± 1.04^a^	5.1 ± 0.12^b^
100µM Nutlin-3a	23.17 ± 1.11^c^	0^c^	62.63 ± 4.17^c^	21.53 ± 1.54^c^	28.33 ± 1.8^c^	35.13 ± 1.84^b^	76.4 ± 4.58^b^	46.02 ± 0.36^b^	2.77 ± 0.17^c^

Spermatozoa kinematics including the percentage of motile spermatozoa (MOT), the percentage of hyper-activated motile spermatozoa (HYP), curvilinear velocity (VCL), straight line velocity (VSL), average path velocity (VAP), linearity (LIN), straightness (STR), wobble (WOB), and lateral head displacement (ALH) were calculated using a CASA system. Data are expressed as the mean ± SEM, n=5. Diﬀerent superscripts (^a,b,c^) within the same row indicate signiﬁcant diﬀerences (*p*< 0.05) on One-way ANOVA.

### Effects of nutlin-3a on sperm capacitation status

**Figure 1:**
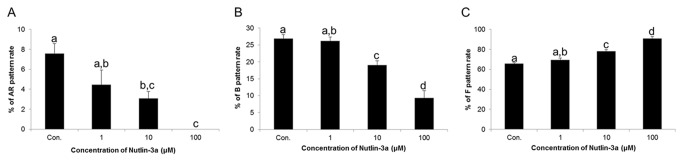
Effects of nutlin-3a on sperm capacitation status *in* *vitro*. (A) Changes in acrosome reacted (AR pattern) spermatozoa in controls and nutlin-3a-treated samples. (B) Changes in capacitated (B pattern) spermatozoa in controls and nutlin-3a-treatedsamples. (C) Changes in non-capacitated (F pattern) spermatozoa in controls and nutlin-3a-treated samples. Data are expressed as the mean ± SEM, n=5. Values with different superscripts (^a,b,c,d^) indicate significant differences between control and treatment groups on one-way ANOVA (*p*<0.05).

 Samples treated with nutlin-3a exhibited a lower acrosome reaction percentage (AR pattern) and decreased capacitation (B pattern) in a dose-dependent manner. However, the percentage of non-capacitated spermatozoa (F pattern) significantly increased with increasing concentrations of nutlin-3a (*p*<0.05). The percentages of samples showing an AR pattern were 7.55 ± 1.01% in control samples and 4.44 ± 1.49 %, 3.07 ± 0.7%, and 0% in 1, 10, and 100μM nutlin-3a-treated spermatozoa, respectively. The percentages exhibiting a B pattern were 26.84 ± 1.26% in controls, 26.13 ± 1.21% in 1μM, 19.02 ± 1.34% in 10μM, and 9.35 ± 2.12% in 100μM nutlin-3a-treated samples. The percentages of F pattern spermatozoa, however, were 65.61 ± 1.08% in controls, 69.42 ± 1.82% in 1μM, 77.91 ± 1.94% in 10μM, and 90.65 ± 2.12% in 100μM nutlin-3a-treated samples (Figure 1).

### Effects of nutlin-3a on change of [Ca^2+^]_i_

**Figure 2:**
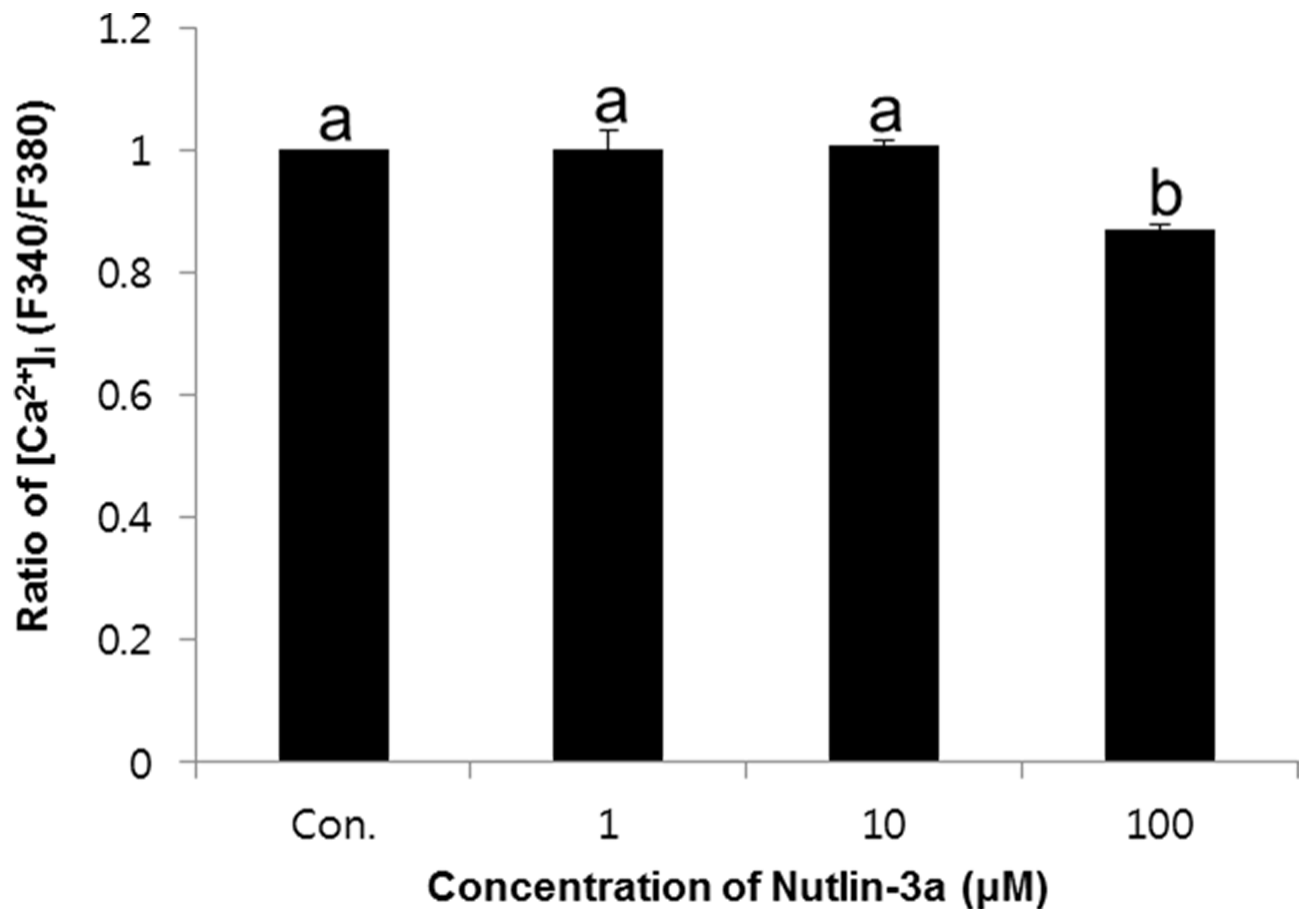
Effects of nutlin-3a on change of [Ca^2+^]_i_ in spermatozoa. Data are expressed as the mean ± SEM, n=3. Values with different superscripts (^a,b^) indicate significant differences between control and treatment groups on one-way ANOVA (*p*<0.05).

The intracellular calcium was detected using [Ca^2+^]_i_ probe Fura-2 AM. The [Ca^2+^]_i_ was expressed as the ratio of the control.[Ca^2+^]_i_ was significantly decreased in the high concentration of nutlin-3a compared with other (1 and 10μM, *p*<0.05) (Figure 2). The ratio of [Ca^2+^]_i_ were 1 ± 0.03 in 1μM, 1.01 ± 0.01 in 10μM, and 0.87 ± 0.01% in 100μM nutlin-3a-treated samples.

### Effects of nutlin-3a on ATP generation

**Figure 3:**
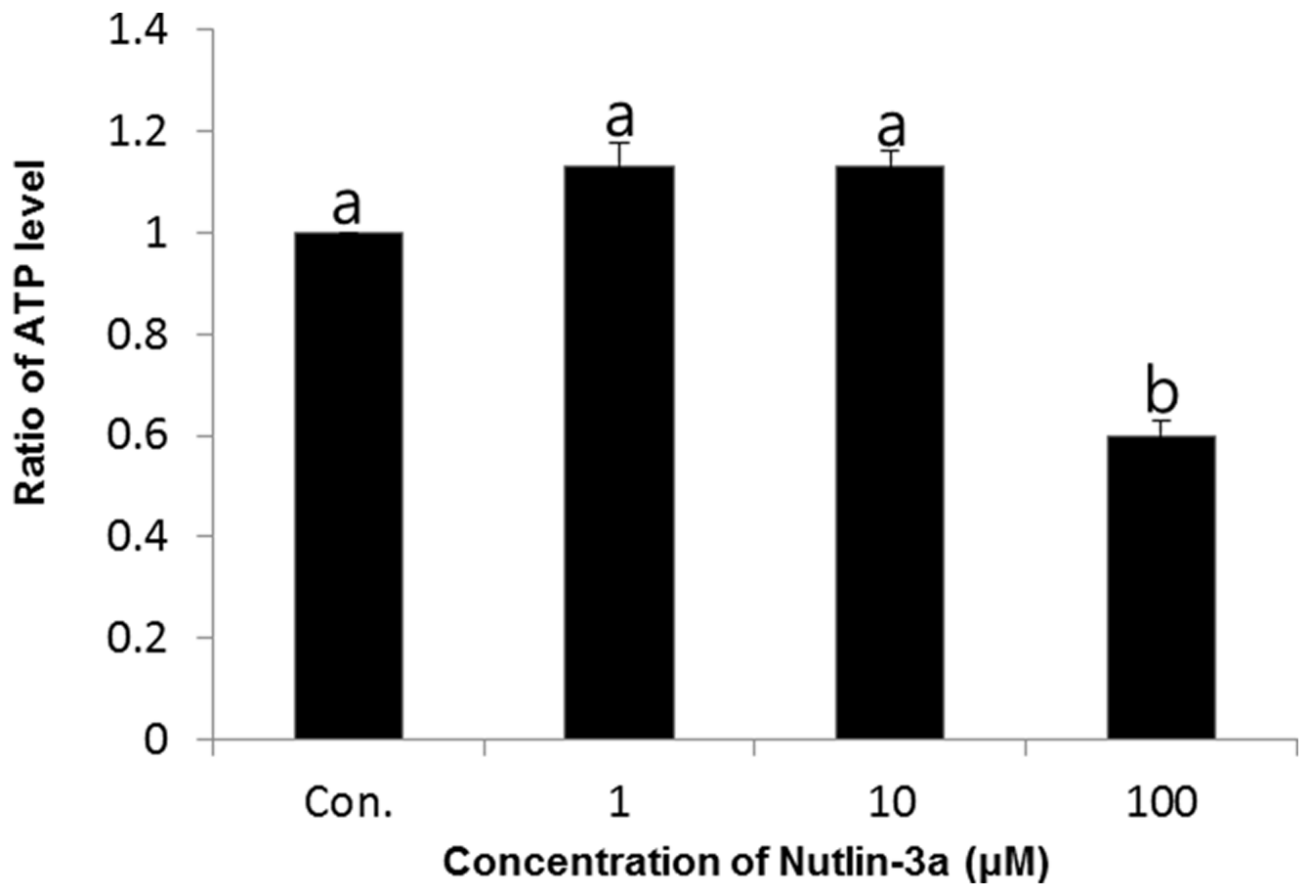
Effects of nutlin-3a on ATP production in spermatozoa. Data are expressed as the mean ± SEM, n=3. Values with different superscripts (^a,b^) indicate significant differences between control and treatment groups on one-way ANOVA (*p*<0.05).

 Quantitative measurements of ATP were performed using a luciferase-driven ATP Bioluminescence Assay Kit. Significantly decreased ATP production was noted in spermatozoa treated with a higher concentration (100μM) of nutlin-3a compared to the other samples (*p*<0.05) (Figure 3). The ratio of ATP was 1.13 ± 0.05 in 1μM, 1.13 ± 0.03 in 10μM, and 0.6 ± 0.03% in 100μM nutlin-3a-treated samples.

### Effects of nutlin-3a on the ubiquinol-cytochrome c reductase core protein II (UQCRC2) and tyrosine phosphorylation proteins

**Figure 4:**
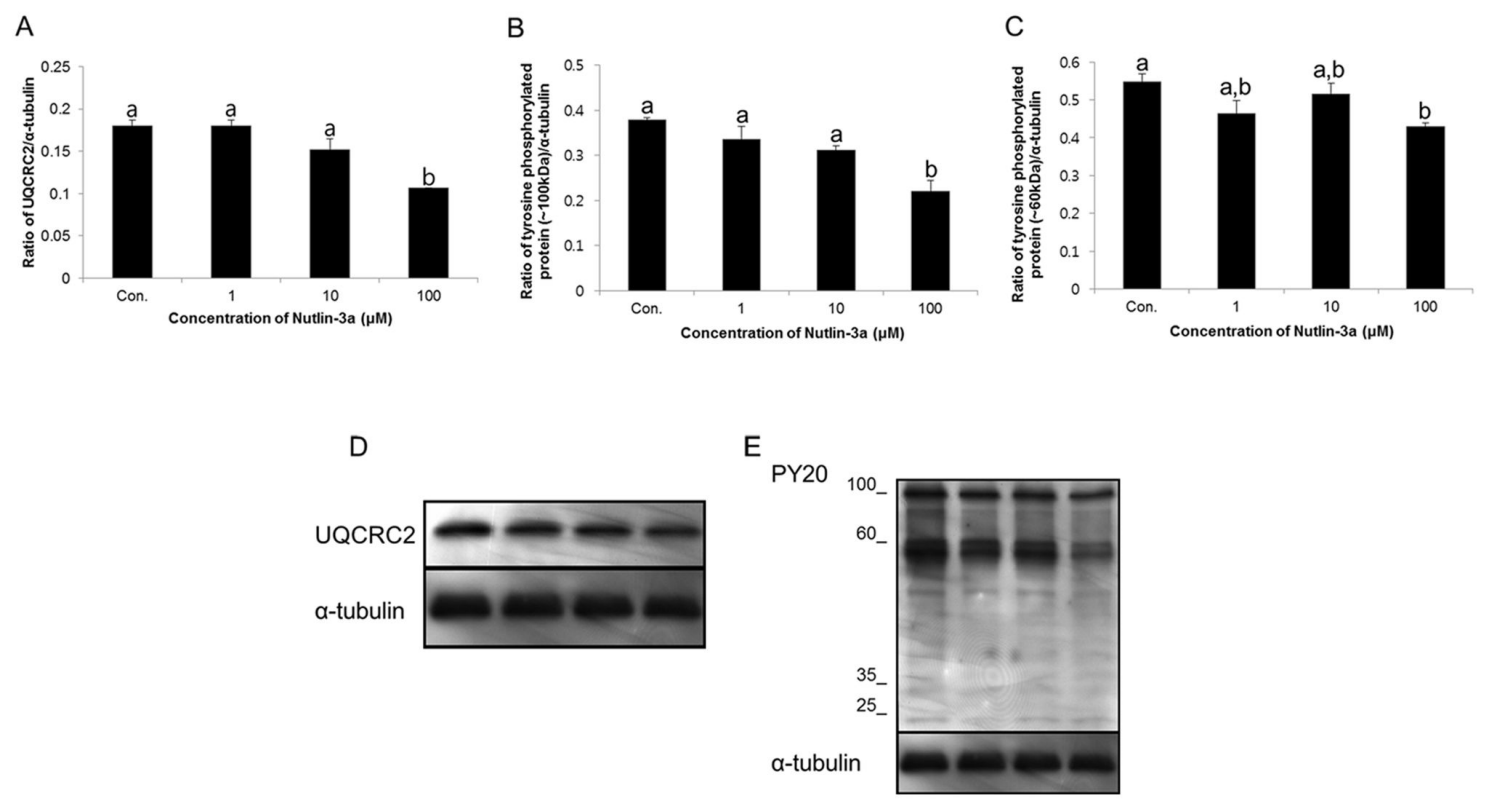
Effects of nutlin-3a on UQCRC2and protein tyrosine phosphorylation. (A) The density of UQCRC2in various treatment groups. Data are expressed as the mean ± SEM, n=3. Values with different superscripts (^a,b^) indicate significant differences between control and treatment groups on one-way ANOVA (*p*<0.05). (B and C) Density of tyrosine phosphorylated protein (~60 and ~100 kDa) in different treatment groups. Data are expressed as the mean ± SEM, n=3. Values with different superscripts (^a,b^) indicate significant differences between control and treatment groups on one-way ANOVA (*p*<0.05). (D) UQCRC2 was probed with anti-UQCRC2 antibody; lane 1=control, lane 2 = 1 µM of nutlin-3a, lane 3 = 10 µM of nutlin-3a, and lane 4 = 100 µM of nutlin-3a. (E) PY 20 was used to probe for tyrosine phosphorylated protein; lane 1 = control, lane 2 = 1 µM of nutlin-3a, lane 3 = 10 µM of nutlin-3a, and lane 4 = 100 µM of nutlin-3a.

 The density of UQCRC2 and tyrosine phosphorylation proteins changed after incubation of spermatozoa with increasing concentrations of nutlin-3a, as shown on western blot analysis. The density of UQCRC2 protein was significantly reduced following treatment with the highest concentration (100 µM) of nutlin-3a (*p*<0.05) (Figure 4A and D). Additionally, tyrosine phosphorylation proteins (~100 and 60 kDa) in spermatozoa treated with the 100 µM of nutlin-3a were significantly reduced compared with the other samples (*p*<0.05) (Figure 4B, C, and E). The density of UQCRC2 was 0.18 ± 0.01% in controls, 0.18 ± 0.01% in 1μM, 0.15 ± 0.01% in 10μM, and 0.11 ± 0.001% in 100μM nutlin-3a-treated samples. The density of tyrosine phosphorylation proteins (~100 kDa) was 0.4 ± 0.004% in controls, 0.34 ± 0.03% in 1μM, 0.31 ± 0.01% in 10μM, and 0.22 ± 0.02% in 100μM nutlin-3a-treated samples. The density of tyrosine phosphorylation proteins (~60 kDa) was 0.55 ± 0.02% in controls, 0.46 ± 0.03% in 1μM, 0.52 ± 0.03% in 10μM, and 0.43 ± 0.01% in 100μM nutlin-3a-treated samples.

### Effects of nutlin-3a on embryonic development and fertilization

**Figure 5:**
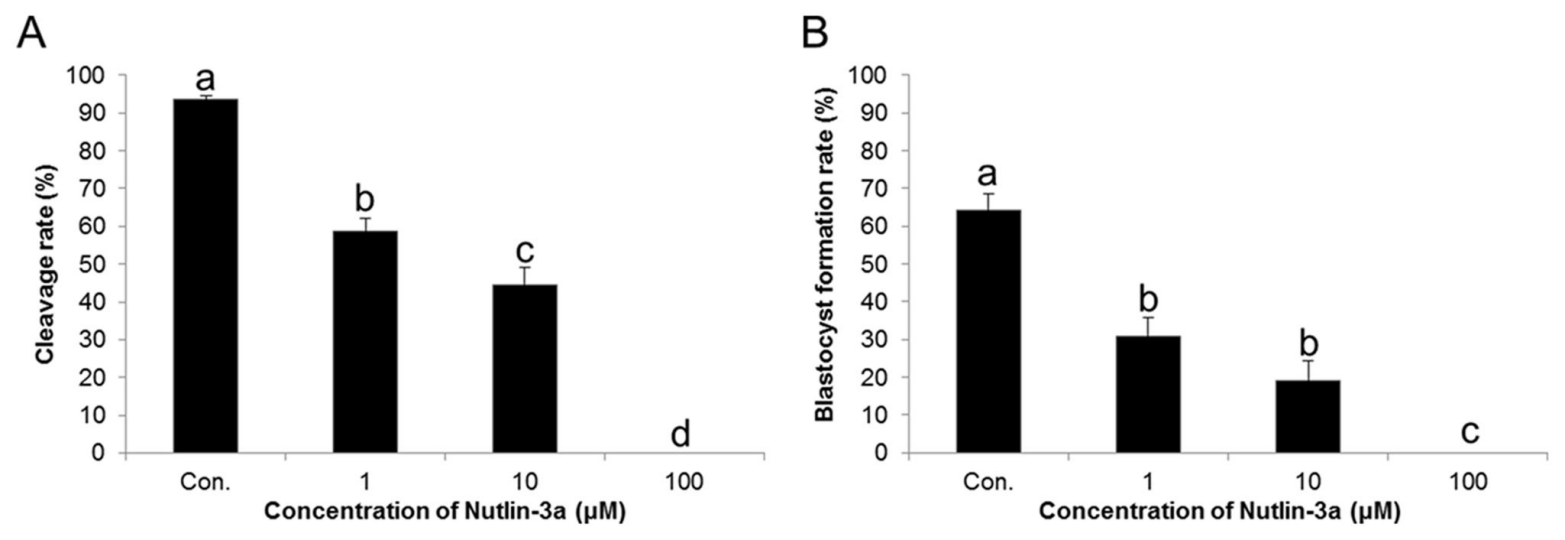
Effects of nutlin-3a on fertilization and embryo development. (A) Fertilization ratesunder different treatment conditions. (B) Blastocyst forming rates under different treatment conditions. Data are expressed as the mean ± SEM, n=4. Values with different superscripts (^a,b,c,d^) indicate significant differences between control and treatment groups on one-way ANOVA (*p*<0.05).

 A significantly decreased cleavage rate was noted in all nutlin-3a-treated groups in a dose-dependent manner (*p*<0.05). The cleavage rates of controls, 1, 10, and 100 µM nutlin-3a-treated spermatozoa samples were 93.61 ± 0.99, 58.82 ± 3.21, 44.44 ± 4.56, and 0, respectively (Figure 5A). However, significant detrimental effects were also observed during embryonic development *in vitro* after treatment with nutlin-3a. The percentages of embryos reaching the blastocyst stage of development were 64.2 ± 4.52% in controls, 30.92 ± 5.02% in 1µM, 19.1 ± 5.18% in 10 µM, and 0% in 100 µM nutlin-3a-treated samples. These effects were dose-dependent (Figure 5B).

## Discussion

 The present study was designed to assess the effects of nutlin-3a on male fertility using mouse spermatozoa. Our results show an important role for nutlin-3a in sperm function via regulation of UQCRC2, TYP as well as associated changes of p53 ubiquitination. We found that nutlin-3a effectively decreased sperm motion kinematics, capacitation, and the acrosome reaction in a dose-dependent manner, with significant negative effects at the highest concentration tested (100 μM). It has been reported in recent studies that nutlin-3a-dependent phosphorylation of p53 either enhances its ability to induce apoptosis or activates pathways that are stimulated in response to cell death or DNA damage [16]. 

 In the current study, decreased expression of UQCRC2 and TYP proteins was noted following the treatment of spermatozoa with higher concentrations of nutlin-3a. Similarly, it has been shown that decreased expression of UQCRC2 is correlated with reduced fertility in bulls [4,17] and increased production of reactive oxygen species (ROS), which has detrimental effects on overall male fertility [18]. Consequently, TYP is associated with capacitation, changes in sperm motility, zona binding competence, and fertilizing ability, whereas the opposite condition had the reverse effect [19]. In particular, sperm TYP and capacitation are both stimulated by cAMP analogues and phosphodiesterase inhibitors, and are inhibited by protein kinase A (PKA) inhibitors, suggesting that cAMP/PKA signaling pathways are involved in these two processes [20].

Currently, we determined that, increased concentration of nutlin-3a was associated with decrease in [Ca^2+^]_i_ (Figure 2) together with UQCRC2 and tyrosine phosphorylation of sperm proteins. These results offer a realistic support that nutlin-3a stimulates ion transport through the sperm membrane. Considering such fact, these observations strongly suggest that spermatozoal ion transport may be partly modulated by nutlin-3a through the inhibition of UQCRC2 with locally released neurotransmitters, and finally contribute to poor fertility. [Ca^2+^]_i_ influx is one of the central biochemical events that occur during functional maturation of sperm cell to fuse with oocyte [21]. There is abundant evidence to support that extracellular [Ca^2+^]_i_ flows into spermatozoa through the CatSper (calcium specific) channel to increase [Ca^2+^]_i_ during capacitation which is predominantly controlled by [Ca^2+^]_i_-ATPase [11,13,22] following increasing the concentration of cAMP [20]. However, [Ca^2+^]_i_ decreases after the morphological maturation of sperm for sperm-oocyte fusion, since acrosome reacted spermatozoa discharge [Ca^2+^]_i_ from inner cells [23].

We evaluated the effects of higher concentrations of nutlin-3a, which was associated with impaired fertilization and embryonic development *in vitro*. These effects may occur due to impaired motility and reduced ATP level, as the ejaculated spermatozoa demand substantial amounts of energy to facilitate *in vivo* transport through the female reproductive tract and for both *in vitro* and *in vivo* penetration of the oocyte zona pellucida [24,25]. Additionally it is important to note that sperm motility relies primarily on the availability of intracellular ATP [26]. A previous report showed that cellular stress induces modifications of both p53 and MDM2 proteins that reduce their activity [27], thereby inhibiting ubiquitination and degradation of the p53 pathway. This may also result in diminished energy for flagellar movement and acrosomal function, which decreases the ability of the spermatozoa to penetrate the oocyte zona pellucida [28,29].

 Additionally, we found that ATP production was decreased in sperm cells treated with nutlin-3a. ATP is essential for the functional and structural integrity of 19S complexes and for the recognition and priming of ubiquitinated proteins destined for proteasomal degradation [30]. ATP is also required for enzymatic reactions during protein ubiquitination, which may occur in spermatozoa during fertilization.

 Nutiln-3a inhibits the activity of p53 by antagonizing MDM2 and it forms auto regulatory feedback loop in which p53 activates MDM2 transcription factor [31,32], and its rapid degradation through the ubiquitin proteolysis pathway. Collectively, our data show that nutlin-3a impairs sperm function due to its effects on sperm kinematics, capacitation, fertilization, and UQCRC2 ubiquitination, and down regulation of ATP and TYP. To our knowledge, this is the first study to investigate the effects of nutlin-3a on mouse spermatozoa. Further studies will be required, however, to validate the true *in vivo* therapeutic potential of nutlin-3a.
